# Highs and lows, ups and downs: Meteorology and mood in bipolar disorder

**DOI:** 10.1371/journal.pone.0173431

**Published:** 2017-03-09

**Authors:** Ben Bullock, Greg Murray, Denny Meyer

**Affiliations:** 1 Department of Psychological Sciences, Swinburne University of Technology, Melbourne, Victoria, Australia; 2 Department of Statistics, Data Science, and Epidemiology, Swinburne University of Technology, Melbourne, Victoria, Australia; Universita Cattolica del Sacro Cuore Sede di Roma, ITALY

## Abstract

Seasonal variation of manic and depressive symptoms is a controversial topic in bipolar disorder research. Several studies report seasonal patterns of hospital admissions for depression and mania and variation in symptoms that appear to follow a seasonal pattern, whereas others fail to report such patterns. Differences in research methodologies, data analysis strategies, and temporal resolution of data may partly explain the variation in findings between studies. The current study adds a novel perspective to the literature by investigating specific meteorological factors such as atmospheric pressure, hours of sunshine, relative humidity, and daily maximum and minimum temperatures as more proximal predictors of self-reported daily mood change in people diagnosed with bipolar disorder. The results showed that daily maximum temperature was the only meteorological variable to predict clinically-relevant mood change, with increases in temperature associated with greater odds of a transition into manic mood states. The mediating effects of sleep and activity were also investigated and suggest at least partial influence on the prospective relationship between maximum temperature and mood. Limitations include the small sample size and the fact that the number and valence of social interactions and exposure to natural light were not investigated as potentially important mediators of relationships between meteorological factors and mood. The current data make an important contribution to the literature, serving to clarify the specific meteorological factors that influence mood change in bipolar disorder. From a clinical perspective, greater understanding of seasonal patterns of symptoms in bipolar disorder will help mood episode prophylaxis in vulnerable individuals.

## Introduction

Many people intuit that bad weather makes us sad and good weather makes us happy. Scientific investigation has largely failed to support such associations however, with variations in meteorological variables either showing no [1, 2] or weak [3] relationships with variations in normal mood. A likely explanation for these weak experimental findings is that weather affects mood for only a subset of the population, and the strength and direction of these effects vary between individuals. Indeed, Klimstra et al. [4] identified four subtypes of people in their Dutch sample according to how individuals’ mood was affected by the weather—Summer Lovers, Summer Haters, Rain Haters, and Unaffected types. The latter subtype, consisting of those that do not experience changes in mood with the weather, was by far the most populated group in the Klimstra et al. sample and goes some way to explaining why average effects of weather on mood across large groups are often not found.

A subset of the population that may be particularly vulnerable to changes in mood due to associated changes in the weather are people diagnosed with bipolar disorder (BD). Unstable mood is a feature of the disorder, even outside the clinical episodes of mania and depression that define the disorder [5], and it has been shown that people with this condition have greater vulnerability to a range of environmental insults (e.g., sleep disturbance, social rhythm disruptions [6]) than the rest of the population. In addition, a seasonal pattern specifier in diagnostic criteria for the disorder (DSM-5 [7]) allows characterisation of clinical mood variation as ‘seasonal’, suggesting that some people with BD present clinically with a repeating pattern of mood disturbance that cycles in a seasonal manner.

Several lines of investigation have been used to systematically evaluate the relationship between season and mood in people with BD. In a comprehensive review of studies across several continents and latitudes, Geoffroy et al. [8] reported peaks in hospital admission rates for depressive episodes in early winter and for manic episodes in spring-summer. A similar seasonal pattern of recurrence was apparent for people diagnosed with the seasonal specifier of BD (approximately 10–15% of all people diagnosed with BD-I). The limitations of data based on hospital admissions records and clinical reports are well-known and significant enough that these findings be interpreted with caution [9]. Geoffroy et al. therefore also reviewed data on the seasonality of self-reported symptoms amongst people with BD and found that about a quarter report a seasonal pattern to their mood symptoms. The most commonly reported pattern across the studies reviewed was a lowering of mood in winter. Similar findings were reported in a long-term prospective study of manic and depressive symptoms conducted over 10 years and based on yearly or half-yearly reports of symptoms to clinicians [10]. While depressive symptoms peaked in winter, particularly amongst those diagnosed with Bipolar I disorder, manic symptoms peaked in autumn, not spring-summer as had previously been reported.

Five further studies investigating prospective relationships between mood and weather in people with BD have failed to find the seasonal patterns reported above. Bauer et al. [9] reported finding no seasonal variation in daily mood self-reports amongst 360 BD outpatients. Friedman et al. [11] found no relationship between clinically-defined depressive episodes and calendar month amongst patients with BD-I enrolled in the Systematic Treatment Enhancement Program for BD (STEP-BD). Christensen et al. [12] investigated seasonal variation via meteorological factors (e.g., temperature, relative humidity, barometric pressure, hours of sunshine), and whether such factors predicted the onset of manic and depressive episodes amongst 56 patients with an ICD-10 diagnosis of Bipolar Affective Disorder over a period of 3 years. Again, no meaningful relationships were found. Finally, Murray et al. [13] reported finding no relationship between season and clinician-rated symptoms of mania or depression amongst 429 patients with BD. Null findings were also reported in a large population-based study of seasonal variation in depressive symptoms [14].

The variation in findings between studies using cross-sectional data such as hospital admission records and those using prospective data from self-reports can probably be explained on methodological grounds. Hospital admission records are low resolution representations of the relationship between season and mood for several reasons—hospital admission dates are often poor indicators of episode onset, dates of episode onset are subject to recall bias, and not all BD patients who experience a mood episode will present to a hospital. Prospective data would appear to be a more authentic way of capturing these relationships, particularly as they pertain to the lived experience of people with BD. If seasonal cycling of symptoms is to be used to facilitate mood episode prophylaxis, then understanding of the lived experience is of paramount importance.

In addition to the methodological importance of prospective self-reports, the use of daily meteorological data to investigate relationships with mood may also confer methodological advantages. As Christensen et al. [12] noted, it is the variation in weather that may explain seasonal influences on mood. Indeed, many of the mechanistic theories used to explain seasonal variation in mood for people with BD are based on variations in daily meteorological factors such as exposure to sunlight and how it affects neurobiological processes [15, 16], rather than the gross and non-specific environmental changes that occur with the seasons. In other words, daily meteorological factors are more *proximal* indicators of how mood is affected by seasonal variation.

**Fig 1:**
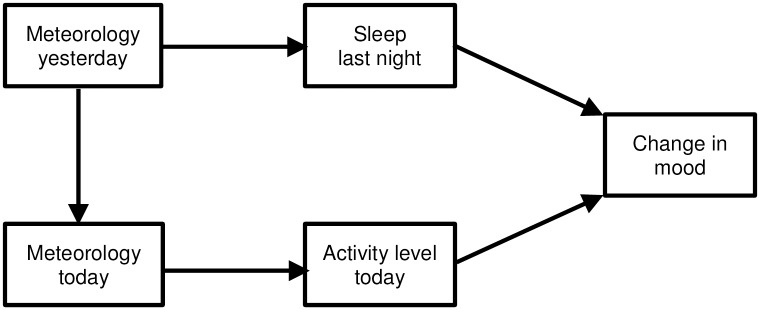
Model of investigated relationships between predictors (meteorology), mediators (sleep, activity), and outcomes (change in mood).

The aim of the current study was to investigate associations between daily mood self-reports and meteorological factors in a sample of people diagnosed with BD. The mixed findings reported in the literature preclude hypotheses pertaining to which meteorological factors, if any, are most important in affecting mood. We also investigated possible mediators of weather-mood relationships in order to better understand *how* these factors might be related. Manifestation of manic and depressive states is associated with higher and lower levels of activity, respectively. It would not be surprising therefore, to see that activity levels partially mediate the weather-mood relationship, as they are also associated with changes in the weather. That is, people tend to be more active in sunny/warmer weather than in darker/colder weather [17]. Sleep-related variables were also investigated as possible mediators of the weather-mood relationship. Fig 1 presents the model of relationships being investigated in the current study.

## Method

### Participants

Eleven participants (8 women, 3 men; age M = 47 years) were recruited through a public health service in regional Victoria, Australia. Inclusion criteria for the study consisted of a primary diagnosis of BD-I using DSM-IV-TR criteria with no significant comorbidity in the form of an Axis-II disorder or a psychotic or substance use disorder. All participants had been clinically stable for at least 6 months at the time of entering the study, and all were taking medications to help manage their condition. Participants maintained a treatment-as-usual approach to the ongoing management of their condition throughout their time in the study. No episodes of mania or depression were reported during participants’ involvement in the study.

An average of 130 consecutive days of self-report data (range: 14 days to 231 days) per participant was available for analysis. Missing observations were found in the mood and sleep data for seven out of the 11 participants. The percentage of missing self-report mood and sleep observations for the whole sample was low however, with only 3.7% of total possible data points not recorded.

### Materials

#### Screening

The Composite International Diagnostic Interview (CIDI-Auto [18]) was used to confirm primary diagnosis. The CIDI-Auto provides a valid and reliable diagnosis of mood disorder based on DSM-IV and ICD-10 criteria.

#### Daily self-report mood recording

ChronoRecord [19] was used by participants to record daily mood and sleep data. Mood was recorded on a 100-unit VAS with user-defined extremes of mania and depression anchoring each end point. ChronoRecord has proven to be a valid and reliable indicator of clinically-relevant mood states in BD [19, 20].

Sleep was also recorded using ChronoRecord. A graphic interface of 24 boxes, each representing one hour in the preceding 24 hours was toggled between three options—Awake, In Bed Asleep, and In Bed Awake. Participants selected the option that best represented their degree of ‘wakefulness’ for each hour in the 24-hour period.

#### 24-hour activity monitoring

Wrist actigraphy was used to record ambulatory 24-hour activity patterns. The actigraph employed in the current study was the Respironics/Mini-Mitter Actiwatch-L (Respironics, Inc., Bend, Oregon) and the Actiware 5.0 software program was used to view and analyse the activity data. The actigraph was worn on the non-dominant wrist of all participants.

#### Sleep

Actigraphy was also used to measure three sleep variables—total sleep time (TST), sleep efficiency (SE), and wake after sleep onset (WASO). TST was calculated as the total number of minutes spent asleep. SE was expressed as the percentage of minutes spent asleep during a given sleep period. A higher percentage indicates more time spent asleep during the sleep period. WASO was defined as the number of minutes spent awake after sleep onset. The use of ChronoRecord afforded the opportunity to collect sleep self-reports alongside these actigraph-recorded sleep variables. Self-reported percentage of time spent in bed actually sleeping (In Bed Asleep) was the variable of interest in the current study.

#### Total daytime activity

A single variable was used to measure total daytime activity. The 10 consecutive hours within the 24-hour day with the highest average level of activity (M10) provides an output variable that can be used to monitor daily activity levels [21].

#### Meteorological data

Meteorological data were obtained from the Australian Bureau of Meteorology’s Climate Data Service (www.bom.gov.au). Daily data pertaining to hours of sunshine, minimum and maximum temperature in degrees Celsius, average relative humidity, and barometric pressure in hectopascals (hPa) were retrospectively matched by date to the mood, sleep, and activity data reported daily by participants. Temperature, humidity, and air pressure data were measured at the airport of the town in regional Victoria from where participants were recruited and where they all resided (latitude 36.8° S). Sunshine data were measured at an international airport, some 150 kilometers from the town in regional Victoria (latitude 37.7° S), because these data were not available from the regional airport.

### Procedure

Participants completed the CIDI-Auto as well as the requisite consent forms. Participants gave written consent to participate in the study and for review of their confidential medical records. Participants received a computer package that included ChronoRecord and other applications necessary to complete and submit mood and sleep reports and were paid for their ongoing participation in the study. Ethical approval for study procedures was gained through both the Swinburne University of Technology Human Research Ethics Committee and the Human Research Ethics Committee of the public health service from which participants were recruited.

### Statistical analysis

In accordance with the mood rating categories for ChronoRecord described by Bauer et al. [19], a mood rating of below 40 was regarded as low mood (depression), above 60 was regarded as high mood (mania) and scores between 40 and 60 were regarded as normal mood. Relationships between mood, weather, activity, and sleep variables were investigated with hierarchical multi-level nominal logistic regression models fitted using HLM7 software [22] to allow for the nested nature of the data within individuals. The sleep variables were added at stage 1, the meteorological variables were added at stage 2, with the M10 activity variable added at Stage 3. All variables were group mean centered prior to analysis in order to avoid potential complications associated with multicollinearity.

In these models the reference category was defined as yesterday’s mood with the probability of transition *i* calculated from the following formula. *No transition* refers to no change in mood between yesterday and today.

$$\text{ln}\left(\frac{P(Transition(i)}{P(No\_transition)}\right)=\beta_{0i}+\beta_{1i}X_{1}+\beta_{2i}X_{2}+\ldots .$$

A mixed linear model analysis was run for the four sleep variables (TST, WASO, SE and In Bed Asleep) in order to determine which of the previous day’s meteorological variables (maximum temperature, difference between maximum and minimum temperature, hours of sunshine, average relative humidity and mean sea level pressure) were related to sleep. This analysis was necessary in order to model possible mediating effects of sleep in the relationship between weather and mood. Several of the sleep variables were transformed in order to make an assumption of normality more plausible for the mixed linear model analysis with a square root transformation for WASO and arcsin(SQRT(y/100)) transformations for SE and In Bed Asleep.

A further mixed linear model analysis was run to determine which of the meteorological variables (maximum temperature, difference between maximum and minimum temperature, hours of sunshine, average relative humidity and mean sea level pressure) were related to activity levels. This analysis was necessary in order to model possible mediating effects of activity in the relationship between weather and mood. In this analysis, participants were regarded as a random factor and an AR dependence structure was assumed for consecutive days.

## Results

Table 1 shows the transition frequencies for mood on consecutive days. Depressed mood days were followed by another depressed mood day for 49.1% of such days with a switch to normal mood for 39.6% of such days and a switch to manic mood for 11.3% of such days. Normal mood days were followed by another normal mood day for 83.4% of such days with a switch to depressed mood for 8.9% of such days and a switch to manic mood for 7.6% of such days. Manic mood days were followed by another manic mood day for 41.1% of such days with a switch to normal mood for 41.7% of such days and a switch to depressed mood for 17.3% of such days. Mood was in the normal range for 70.7% of all days.

**Table 1:** Observed upward and downward mood transitions on consecutive days.

Mood Yesterday; *n* days (%)				
Mood Today	Depressed	Normal	Manic	Total
Depressed	113 (49.1)	84 (8.9)	29 (17.3)	226 (16.9)
Normal	91 (39.6)	786 (83.4)	70 (41.7)	947 (70.7)
Manic	26 (11.3)	72 (7.6)	69 (41.1)	167 (12.5)
Total	230 (100)	942 (100)	168 (100)	1340 (100)

### Effect of last night’s sleep, today’s meteorological variables and today’s activity levels on changes in mood

Relationships between variables were tested using nominal logistic regression analyses. Separate analyses were performed for depressed, normal and manic mood yesterday, allowing yesterday’s mood to moderate the relationships with changes in mood. Each analysis was carried out using multi-level modelling in three stages as previously described. In these multinomial logistic regression analyses the condition of no mood change from yesterday was considered the reference category. The first analyses, summarised in Tables 2 and 3, consider shifts in mood when yesterday’s mood was depressed.

**Table 2:** Sleep, activity, and meteorological variables associated with changes from depressed mood yesterday to normal mood today.

	Stage 1		Stage 2		Stage 3	
	Odds Ratio	95% CI	Odds Ratio	95% CI	Odds Ratio	95% CI
TST	1.000	(.995,1.004)	.999	(.995,1.005)	1.000	(.996,1.005)
SE	.939	(.864,1.021)	.938	(.859,1.024)	.930	(.845,1.025)
WASO	.992	(.976,1.009)	.990	(.973,1.007)	.989	(.971,1.007)
In Bed Asleep	1.042	(.999,1.087)	1.050*	(1.004,1.009)	1.048*	(1.002,1.097)
Max Temperature			1.036	(.936,1.146)	1.021	(.921,1.133)
Max-Min Temperature			1.098	(.991,1.217)	1.103	(.994,1.223)
Hours Sunshine			1.006	(.895,1.131)	.998	(.886,1.125)
Average Relative Humidity			1.012	(.969,1.057)	1.008	(.964,1.054)
Mean Sea Level Pressure			.947	(.895,1.003)	.942*	(.889,.997)
M10					.998	(.994,1.003)

*Note*. TST = Total Sleep Time (total number of minutes spent asleep per day), SE = Sleep Efficiency (percentage of minutes spent asleep during a sleep period), WASO = Wake After Sleep Onset (number of minutes spent awake after sleep onset), In Bed Asleep (self-reported percentage of time spent in bed actually sleeping), M10 = Total daytime activity derived from the 10 consecutive most active hours in the day.

*p<.05

**Table 3:** Sleep, activity, and meteorological variables associated with changes from depressed mood yesterday to manic mood today.

	Stage 1		Stage 2		Stage 3	
	Odds Ratio	95% CI	Odds Ratio	95% CI	Odds Ratio	95% CI
TST	1.003	(.997,1.009)	1.003	(.997,1.009)	1.004	(.997,1.010)
SE	.932	(.853,1.017)	.945	(.860,1.037)	.924	(.833,1.026)
WASO	.974	(.949,1.001)	.964*	(.936,.994)	.964*	(.935,.993)
In Bed Asleep	.984	(.903,1.073)	1.005	(.916,1.101)	1.009	(.918,1.109)
Max Temperature			1.190*	(1.035,1.368)	1.175*	(1.022,1.351)
Max-Min Temperature			.966	(.827,1.128)	.981	(.839,1.148)
Hours Sunshine			1.190	(.979,1.447)	1.167	(.955,1.427)
Average Relative Humidity			1.051	(.987,1.120)	1.046	(.982,1.114)
Mean Sea Level Pressure			1.038	(.949,1.135)	1.030	(.941,1.128)
M10					1.004	(.996,1.012)

*Note*. TST = Total Sleep Time (total number of minutes spent asleep per day), SE = Sleep Efficiency (percentage of minutes spent asleep during a sleep period), WASO = Wake After Sleep Onset (number of minutes spent awake after sleep onset), In Bed Asleep (self-reported percentage of time spent in bed actually sleeping), M10 = Total daytime activity derived from the 10 consecutive most active hours in the day.

*p<.05

Table 2 shows that In Bed Asleep had a significant positive relationship with changes from depressed to normal mood. On average, the odds of a switch from depressed to normal mood increased by 5% for a 1% increase in the percentage of time spent in bed asleep. A significant relationship between Mean Sea Level Pressure and a switch from depressed to normal mood was also found. On average, a 1 hPa increase in Mean Sea Level Pressure was associated with a 6% reduction in the odds of a switch from depressed to normal mood.

Table 3 shows that there was a significant relationship between maximum temperature and the odds of a transition from depressed to manic mood. A 1° increase in the maximum temperature was associated with a 17.5% increase in the odds of a transition from depressed to manic mood on average. The number of minutes spent awake after sleep onset was also significantly associated with the odds of a transition from depressed to manic mood. For each additional minute awake the odds of a shift from depressed to manic mood declined by 3.6% on average.

In the next analysis, no significant predictors were found for transitions from normal to manic mood. However, as shown in Table 4, there were some significant predictors for transitions from normal to depressed mood.

**Table 4:** Sleep, activity, and meteorological variables associated with changes from normal mood yesterday to depressed mood today.

	Stage 1		Stage 2		Stage 3	
	Odds Ratio	95% CI	Odds Ratio	95% CI	Odds Ratio	95% CI
TST	1.000	(.996,1.003)	.999	(.995,1.003)	.998	(.995,1.002)
SE	1.079*	(1.009,1.155)	1.073*	(1.003,1.149)	1.077*	(1.005,1.155)
WASO	.997	(.984,1.010)	.999	(.986,1.012)	.999	(.986,1.012)
In Bed Asleep	.972	(.945,1.000)	.971*	(.943,1.000)	.970*	(.942,.999)
Max Temperature			.990	(.915,1.070)	.968	(.899,1.042)
Max-Min Temperature			.948	(.875,1.027)	.964	(.896,1.039)
Hours Sunshine			1.017	(.933,1.109)	1.026	(.941,1.118)
Average Relative Humidity			1.015	(.986,1.045)	1.014	(.985,1.044)
Mean Sea Level Pressure			1.017	(.978,1.058)	1.024	(.983,1.065)
M10					.996**	(.993,.998)

*Note*. TST = Total Sleep Time (total number of minutes spent asleep per day), SE = Sleep Efficiency (percentage of minutes spent asleep during a sleep period), WASO = Wake After Sleep Onset (number of minutes spent awake after sleep onset), In Bed Asleep (self-reported percentage of time spent in bed actually sleeping), M10 = Total daytime activity derived from the 10 consecutive most active hours in the day.

*p<.05

**p<.01

The data in Table 4 suggest that, on average, the odds of a transition from normal to depressed mood increased by 7.7% for each additional 1% of SE, but declined by 3.0% for each additional 1% of time spent in bed asleep. In addition, there was a significant association with M10, with the odds of a transition from normal to depressed mood on average declining by 0.4% for each additional unit increase in M10 raw activity.

Finally, transitions from manic mood were investigated. No significant predictors were found for transitions from manic to depressed mood. However, Table 5 shows a single significant predictor—In Bed Asleep—for transition from manic to normal mood. A transition from manic to normal mood was more likely after a good night’s sleep. For each 1% increase in time spent in bed asleep the odds of a transition from manic to normal mood increased by 14.8% on average.

**Table 5:** Sleep, activity, and meteorological variables associated with changes from manic mood yesterday to normal mood today.

	Stage 1		Stage 2		Stage 3	
	Odds Ratio	95% CI	Odds Ratio	95% CI	Odds Ratio	95% CI
TST	.997	(.991,1.003)	.997	(.991,1.003)	.997	(.990,1.003)
SE	1.014	(.971,1.059)	1.014	(.971,1.058)	1.042	(.976,1.113)
WASO	1.007	(.989,1.025)	1.007	(.989,1.025)	1.009	(.990,1.029)
In Bed Asleep	1.137**	(1.049,1.233)	1.141**	(1.050,1.240)	1.148**	(1.053,1.252)
Max Temperature			.973	(.865,1.095)	.976	(.865,1.102)
Max-Min Temperature			.964	(.837,1.111)	.979	(.848,1.130)
Hours Sunshine			1.036	(.889,1.208)	1.010	(.866,1.180)
Average Relative Humidity			.983	(.940,1.029)	.983	(.938,1.030)
Mean Sea Level Pressure			1.014	(.941,1.092)	1.017	(.942,1.096)
M10					1.000	(.996,1.005)

*Note*. TST = Total Sleep Time (total number of minutes spent asleep per day), SE = Sleep Efficiency (percentage of minutes spent asleep during a sleep period), WASO = Wake After Sleep Onset (number of minutes spent awake after sleep onset), In Bed Asleep (self-reported percentage of time spent in bed actually sleeping), M10 = Total daytime activity derived from the 10 consecutive most active hours in the day.

*p<.05

**p<.01

Overall, the results show that higher maximum temperatures were associated with transition from depressed to manic mood while higher mean sea level pressure appeared to inhibit a transition from depressed to normal mood. Increased M10 activity apparently only protected against transitions from normal to depressed mood. A higher percentage of time spent in bed asleep was associated with increased odds of a transition from depressed to normal mood and from manic to normal mood, with decreased odds of a transition from normal to depressed mood, and thus suggest that percentage of time spent in bed asleep had a protective effect on unhealthy mood transitions. Additional minutes awake after sleep onset appeared to reduce the odds of a transition from depressed to manic mood while increased sleep efficiency appeared to increase the odds of a transition from normal to depressed mood.

### Effect of meteorological variables on sleep

A mixed linear model analysis was run for the four sleep variables (TST, WASO, SE and In Bed Asleep) in order to determine which of the previous day’s meteorological variables were related to sleep (Table 6).

**Table 6:** Mixed linear meteorological models for sleep.

Yesterday’s Meteorological Conditions	t-statistics			
	WASO†	In Bed Asleep††	SE††	TST
Hours Sunshine	.217	-.141	.754	.943
Max Temperature	-1.404	-2.604**	-1.746	-2.693**
Max-Min Temperature	.493	.450	1.370	1.448
Average Relative Humidity	-1.498	.756	-1.752	-1.728
Mean Sea Level Pressure	-.152	-.417	-.741	-.621

*Note*. TST = Total Sleep Time (total number of minutes spent asleep per day), SE = Sleep Efficiency (percentage of minutes spent asleep during a sleep period), WASO = Wake After Sleep Onset (number of minutes spent awake after sleep onset), In Bed Asleep (self-reported percentage of time spent in bed actually sleeping).

^†^SQRT(Y) transformation

^††^arcsin(SQRT(Y/100)) transformation

**p<.01

The results show that maximum temperature was the only meteorological variable that had a significant linear relationship with In Bed Asleep and TST, with higher temperatures being associated with less sleep.

In combination with the significant relationships between last night’s sleep and today’s mood, these findings suggest that the effect of meteorological variables on transitions in mood may be partially mediated by the effect of meteorological variables on sleep.

### Effect of today’s meteorological variables on activity levels

A mixed linear model analysis was then run in order to determine whether today’s activity levels were related to the meteorological variables. Table 7 suggests that hours of sunshine and mean sea level pressure have a positive relationship with activity levels on the same day. It is possible then, that activity levels partially mediate the effect of meteorological variables on transitions in mood.

**Table 7:** Mixed linear meteorological model for activity level.

Today’s Meteorological Conditions	t-statistics M10
Maximum Temperature	-1.224
Max-Min Temperature Difference	.682
Hours of Sunshine	2.105*
Average Relative Humidity	-.386
Mean Sea Level Pressure	2.100*

M10 = Total daytime activity derived from the 10 consecutive most active hours in the day.

*p<.05

## Discussion

Overall, the results supported effects of two meteorological variables on mood. Specifically, higher maximum temperature was associated with an increase in the odds of a transition from depressed mood to manic mood on consecutive days. In addition, improvement in mood from depressed to normal on consecutive days was more likely the lower the recorded sea-level pressure was on that day. Effects of sunshine hours, humidity, and minimum temperature on transitions in mood were not found.

That higher maximum temperatures were associated with transitions from depressed to manic mood on consecutive days is a novel finding, particularly in the absence of an effect of sunshine hours on mood—an effect that may have been expected based on theory. Previous data have shown that sunshine can have a positive effect on mood [4], and theory would suggest that increased hours of access to sunshine (or alternative sources of bright light [15]) during the day would have a stabilising effect on neurobiological systems that are relevant to mood disorders [16]. No such data exists for an independent effect of temperature on mood.

A transition in mood of the magnitude recorded may be clinically significant, as it indicates a rapid change from a depressed mood state to a manic one. Such rapid changes in mood are indicative of worse outcomes in BD populations [23]. Notably, the significant effect was isolated to instances in which mood transitioned upwards from depressed to manic (*n* = 29), a transition which is considered a *worsening* of mood. Maximum temperature was not associated with a transition from normal mood to manic (*n* = 72), or from depressed to normal mood (*n* = 91), the latter of which in particular may be considered an *improvement* in mood.

The relationship between mood and sea-level pressure was also a novel finding. No previous study has demonstrated any effect, positive or negative, of atmospheric pressure on mood. Theories on why such an effect might exist are similarly non-existent. The magnitude of the relationship between lower sea-level pressure and improvement in mood from depressed to normal in the current study was very small—a 1 hPa decrease in Mean Sea Level Pressure was associated with only a 6% increase in the odds of a switch from depressed to normal mood. The potential clinical relevance of such a small effect is arguably moot. In the absence of any practical or theoretical justification for such a relationship, this finding must be considered questionable until replicated.

Stronger relationships were reported for the effects of sleep on mood. In particular, self-reported percentage of time spent in bed asleep was associated with greater odds of transition into normal mood, from both depressed and manic mood the previous day, and lower odds of a transition from normal mood the previous day into depressed mood the next day. It would seem therefore, that longer self-reported time spent in bed asleep had a protective effect on unhealthy mood transitions in this sample of people with BD-I. The significant associations between sleep and mood were not consistently replicated using actigraph-derived objective estimates of sleep, perhaps reinforcing the fundamental differences between, and inherent limitations of, these two measures of sleep, particularly in populations with disturbed sleep [24]. Nevertheless, the importance of adequate sleep in managing BD is unquestionable [25] and the current findings appear to support clinical recommendations.

Based on the results reported here it is possible that any meteorological effects on mood are, at least in part, mediated by sleep and activity variables. The findings in the current study that specific meteorological factors can affect sleep (Table 6) and activity (Table 7), when considered alongside the well-characterised associations between mood and both sleep [26] and activity [27] in BD populations, suggest a hypothesised mediational relationship in the direction of meteorological factors -> sleep/activity -> mood. Further direct evidence of such a causal pathway is needed.

A potential mediator of the relationship between meteorological factors and mood not investigated in the current study is seasonal variation in social interaction (i.e., greater in Summer, lesser in Winter). Such variation in social interaction may have particular relevance for people with BD [28, 29]. The number and valence of social interactions should be measured in future studies of weather-mood relationships in BD populations.

The small sample size and the fact that all participants were taking medication to manage their BD are limitations of the current study. The possibility that the reported outcomes are idiosyncratic to this sample can not be discounted. In addition, obtaining data on individuals’ actual exposure to natural light, rather than relying on meteorological reports of the amount of sunshine hours per day, would be desirable methodological inclusions in future studies. Similarly, self-reports of mood, particularly manic mood, may need to be corroborated with others’ reports of participants’ mood given the lack of insight often associated with these mood states in people with BD [30]. Given these limitations, the findings are undoubtedly preliminary and exploratory in nature. Replication in larger and more diverse samples is necessary.

The outcomes of the current study provide a novel perspective on the questions surrounding seasonal mood patterns in BD. In particular, we were interested in the specific meteorological factors (if any) that affect mood in this population, and possible mediators of these relationships. The data supported a small effect of temperature on mood in this sample, with higher maximum temperature being associated with worsening of mood towards mania. This effect of temperature was likely mediated by sleep. The lack of consensus in the BD literature on weather-mood relationships is largely due to variations in how mood disorder states are measured and conceptualised in BD (episodes, hospitalisation, self-reports) and the most appropriate time lag to monitor these relationships (synchronous, previous day, weekly, monthly). The current study investigated the relationship between meteorological factors and mood at a more proximal level than previous investigations by using daily and more specific meteorological data as well as daily reports of mood, and thus makes an important contribution to our understanding of seasonal mood patterns in BD. A more nuanced understanding of meteorological influences on mood in BD will assist clinical- and self-management of manic and depressive episodes that recur with the seasons.
